# Sodium bicarbonate supplementation improves severe-intensity intermittent exercise under moderate acute hypoxic conditions

**DOI:** 10.1007/s00421-018-3801-7

**Published:** 2018-01-17

**Authors:** Sanjoy K. Deb, Lewis A. Gough, S. Andy Sparks, Lars R. McNaughton

**Affiliations:** 1 Sports Nutrition and Performance Research Group, Department of Sport and Physical Activity, Edge Hill University, Ormskirk, Lancashire L39 4QP UK; 2 Department of Sport and Movement Studies, Faculty of Health Science, University of Johannesburg, Johannesburg, South Africa

**Keywords:** Alkalosis, Altitude, Extreme environments, Intermittent hypoxic exercise, Critical power, Severe-intensity domain

## Abstract

Acute moderate hypoxic exposure can substantially impair exercise performance, which occurs with a concurrent exacerbated rise in hydrogen cation (H^+^) production. The purpose of this study was therefore, to alleviate this acidic stress through sodium bicarbonate (NaHCO_3_) supplementation and determine the corresponding effects on severe-intensity intermittent exercise performance. Eleven recreationally active individuals participated in this randomised, double-blind, crossover study performed under acute normobaric hypoxic conditions (FiO_2_% = 14.5%). Pre-experimental trials involved the determination of time to attain peak bicarbonate anion concentrations ([HCO_3_^−^]) following NaHCO_3_ ingestion. The intermittent exercise tests involved repeated 60-s work in their severe-intensity domain and 30-s recovery at 20 W to exhaustion. Participants ingested either 0.3 g kg bm^−1^ of NaHCO_3_ or a matched placebo of 0.21 g kg bm^−1^ of sodium chloride prior to exercise. Exercise tolerance (+ 110.9 ± 100.6 s; 95% CI 43.3–178 s; *g* = 1.0) and work performed in the severe-intensity domain (+ 5.8 ± 6.6 kJ; 95% CI 1.3–9.9 kJ; *g* = 0.8) were enhanced with NaHCO_3_ supplementation. Furthermore, a larger post-exercise blood lactate concentration was reported in the experimental group (+ 4 ± 2.4 mmol l^−1^; 95% CI 2.2–5.9; *g* = 1.8), while blood [HCO_3_^−^] and pH remained elevated in the NaHCO_3_ condition throughout experimentation. In conclusion, this study reported a positive effect of NaHCO_3_ under acute moderate hypoxic conditions during intermittent exercise and therefore, may offer an ergogenic strategy to mitigate hypoxic induced declines in exercise performance.

## Introduction

Acute ambient hypoxic environments are often used as an ergogenic strategy to enhance exercise-induced training adaptations (Lundby et al. 2012). Indeed, methods that involve interspersed acute hypoxic exercise bouts within a training programme, are suggested to augment molecular training adaptations leading to enhanced anaerobic glycolytic activity (Faiss et al. 2013). This benefit is not without cost however, as the lower availability of oxygen (O_2_) may elicit an ergolytic effect on exercise intensity and volume during intermittent and continuous exercise (Aldous et al. 2016; Clark et al. 2007). The precise reasons causing these attenuations in exercise performance are ambiguous; however, an integrated central and peripheral fatigue response is likely (Fan and Kayser 2016). This includes a lower convective O_2_ delivery to active skeletal muscle (Amann and Calbet 2008), an exacerbated disturbance to acid–base balance during exercise (Hogan et al. 1999; Romer et al. 2007) and under severe hypoxic conditions (> 3000 m), a further reduction in group III/IV afferent feedback to diminish central motor output is apparent (Amann et al. 2007). The resultant decline in exercise performance presents a challenge to the management of training load during acute hypoxic training regimes to ensure the acute cost to exercise performance does not hamper the potential medium to long-term benefits of these strategies.

Acute dietary strategies have previously been used to mitigate for the impaired exercise performance caused by acute hypoxia. This includes dietary nitrate supplementation to enhance convective O_2_ delivery (Shannon et al. 2017) and sodium bicarbonate (NaHCO_3_) supplementation as an alkalotic buffer to dampen the elevated acidic stress (Deb et al. 2017). The latter presents an interesting physiological paradigm, given the relative increase in glycolytic flux with hypoxic exposure potentiating hydrogen cation (H^+^) production. However, their concurrent removal may be hindered as blood bicarbonate buffering capacity may be diminished under hypoxic conditions, due to a suggested lower bicarbonate anion concentrations ([HCO_3_^−^]) (Cerretelli and Samaja 2003). It is therefore intuitive to assess ergogenic strategies that may facilitate the removal of excess H^+^ during exercise and compensate for the suggested [HCO_3_^−^] reductions. Indeed, Deb et al., (2017) reported that the efficacy of NaHCO_3_ supplementation is enhanced under acute hypoxia compared to sea level as the magnitude of improvement was greater during high-intensity exercise in acute hypoxic conditions. This should be interpreted with caution however, given that previous studies have either reported no benefit with NaHCO_3_ under acute hypoxic conditions (Saunders et al. 2014a; Flinn et al. 2014), or inconsistencies in the overall ergogenic response (Froio de Araujo Dias et al. 2015). Furthermore, there remains considerable contention on the importance of the acid–base balance on fatigue and exercise performance, as exercise performance can be maintained despite perturbations in acid–base balance (Fitts 2016; Westerblad 2016). Consequently, further research is required to elucidate the importance of the acid–base balance, particularly under acute hypoxic exposures where pre-exercise alkalotic manipulation may induce beneficial performance outcomes for isolated exercise bouts.

It is evident through viewing blood lactate kinetics during exercise, and corresponding disturbances in acid–base balance, that the ergogenic effects of NaHCO_3_ may only arise during exercise intensities at or above the severe-intensity domain. This cluster of exercise intensities can be distinguished by physiological markers defined by the second lactate and ventilatory (or otherwise known as the respiratory compensation point) thresholds, or critical power at the lower boundary, whilst the upper boundary is defined as the intensity at the peak rate of oxygen consumption ($\dot {V}$O_2peak_) (Jones et al. 2010). Within this given intensity range, an inexorable rise in lactate occurs and the acid–base balance becomes substantially perturbed to performance limiting levels (Jones et al. 2007). Indeed, work performed in the severe-intensity domain during continuous exercise is enhanced with prior NaHCO_3_ supplementation (Egger et al. 2014); however, this is not reflected in exercise tolerance during severe-intensity intermittent exercise under hypoxic conditions in acclimatised individuals (Kozak-Collins et al. 1994). The acclimatised participants used in the latter study may explain the lack of effect, given altitude acclimatisation negates the additional acidic load apparent in acclimatised individuals under acute hypoxia (West 2007). As the ergogenicity of NaHCO_3_ is dependent on the magnitude of acid–base perturbations, it is hypothesised that NaHCO_3_ supplementation will improve severe-intensity intermittent exercise under acute hypoxic conditions.

## Method

Eleven recreationally active male volunteers (see Table 1 for participant characteristics), with no sustained altitude exposure in the preceding 6 months, participated in this investigation. All participants performed regular physical exercise and were also accustomed to repeated high-intensity intermittent cycling exercise. This included eight participants that regularly partook in cycling exercise (> 60 km week^−1^ and > 7 h week^−1^), which is in accordance with the training volume that classifies individuals as trained (De Pauw et al. 2015), whilst the remaining three participants performed cycling activity as part of a regular exercise regime (≥ 4 h week^−1^), which represents volume of work that classifies individuals as recreationally active individuals (De Pauw et al. 2015). Prior to obtaining written consent, participants were informed of the purpose, benefits and risks of participation. Ethical approval was attained from the institutional ethics committee and conducted in accordance with the Helsinki Declaration. Participants were instructed to refrain from strenuous exercise and alcohol consumption in the preceding 24 h before each laboratory visit; while also abstaining from caffeine for 12 h. A 24-h dietary recall was completed during the initial visit, with participants asked to replicate dietary intake for subsequent visits. This was confirmed verbally on arrival to each laboratory visit. Participants arrived in a 3-h postprandial state and were asked to maintain water intake prior to arrival to limit confounding nutritional effects on exercise performance.

**Table 1:** Participant characteristics

Variables	Hypoxia
Age	28 ± 6
Height (cm)	179.9 ± 7.2
Weight (kg)	81.7 ± 11.8
BMI	25.2 ± 2.6
Peak power output (W)	333 ± 46
$\dot {V}$O_2peak_ (l min^−1^)	3.3 ± 0.4
Ventilatory threshold 1 (W)	159 ± 27
Critical power (W)	222 ± 33
*W*ʹ (kJ)	21.3 ± 4.5

### Experimental design

A randomised, double-blind, crossover experimental design was employed with participants attending the laboratory up to six separate occasions at the same time of day (± 1 h). All exercise trials were a minimum 24 h apart and completed within a 3-week period. A normobaric environmental chamber (Model S016r-7-sp, TISS, Portsmouth, UK) was used to recreate ambient hypoxic conditions with a fractional inspired O_2_ percentage (FiO_2_%) of 14.5%; while temperature (20 °C) and humidity (40%) was also regulated throughout the study. Participants were exposed to hypoxic air 10 min prior to exercise to allow equilibrium between atmospheric and body O_2_ stores (Andreassen and Rees 2005).

During the initial visit, individual blood acid–base response to 0.3 g kg^−1^ body mass of NaHCO_3_ supplementation was established through measuring the time course of blood acid–base balance across a 90-min period following ingestion (Gough et al. 2017). Sodium bicarbonate was administered in 400 ml of chilled water and mixed with 50 ml of sugar-free cordial (blackcurrant squash, Heritage, UK); with the participants asked to consume the supplement within a 10-min time period. Fingertip capillary blood samples (70 µl) were drawn every 10 min for 60 min and then every 5 min from 60 to 90 min. Participants remained seated during the collection of blood into a capillary tube (Electrolyte balanced heparin clinitube, Radiometer, Denmark). Blood samples were analysed for [HCO_3_^−^] using a blood gas analyser (Radiometer ABL800, Denmark). The individual time taken for peak [HCO_3_^−^] to occur was then used for the pre-ingestion timing for subsequent experimental exercise trials. This method controls for the intra-individual differences in acid–base kinetics following NaHCO_3_ supplementation (Jones et al. 2016; Gough et al. 2017) and enables individuals to exercise at their peak blood [HCO_3_^−^] to maximise the HCO_3_^−^ buffering potential. This timeframe in attaining peak blood [HCO_3_^−^] following NaHCO_3_ is shown to be reproducible (*r* = 0.94; *p* < 0.001) between ingestions within participants (Gough et al. 2017) and therefore, represents a reliable method to administer NaHCO_3_.

Following the first visit to establish the timeframe for attaining peak [HCO_3_^−^], baseline $\dot {V}$O_2peak_ and the ventilatory threshold 1 (VT1) were determined on the second laboratory visit using an incremental RAMP test performed under simulated hypoxic conditions (FiO_2_% = 14.5%). This was followed by familiarisation to the 3-min all-out test after 30-min recovery from the RAMP test. Subsequently, on separate laboratory visits, participants performed the 3-min test on a further two occasions under acute hypoxic conditions. However, if a valid test was not completed (described in further detail below), participants would repeat the test during additional laboratory visits. The final two visits involved an exhaustive intermittent exercise test performed under two different randomised experimental conditions, with the prior ingestion of either 0.3 g kg bm of NaHCO_3_ or placebo containing 0.21 g kg bm of sodium chloride (NaCl). The placebo was also mixed in 400 ml of water and 50 ml of cordial to mask the experimental conditions; while the dose of NaCl was chosen as it presents an equimolar composition of sodium to 0.3 g kg bm of NaHCO_3_, which consequently mitigates for the potential ergogenic effects of sodium (Mora-Rodriguez and Hamouti 2012).

### Determination of $\dot {V}$O_2peak_ and ventilatory threshold1

The RAMP test commenced with 3-min unloaded pedalling into a ramped increase of 1 W s^−2^, equivalent to 30 W min^−1^. A preferred cadence was selected prior to the test, which participants were asked to maintain until volitional exhaustion. The test was terminated when this cadence could no longer be maintained within 10 rpm for 10 s, despite strong verbal encouragement. Exercise tests were performed on an electrically braked ergometer (Lode Excalibur Sport, Groningen, The Netherlands), with the frame dimensions and pedals adjusted to participant preference and replicated for all subsequent exercise trials. Breath-by-breath pulmonary gas exchange was recorded throughout, and in all subsequent trials, using a metabolic analyser (Oxycon, Jaeger, Germany). Peak power output (PPO) was defined as the greatest power output attained at the termination of the test, whilst $\dot {V}$O_2peak_ was defined as the highest 30 s rolling average of $\dot {V}$O_2_. Ventilatory threshold 1 (VT1) was estimated using the detection of inflection points on gas exchange graphs as outlined by Beaver et al. (1986).

### Three-min critical power test

A 3-min test was used to determine critical power (CP) and *W* prime (*W*ʹ) to inform the work intensity used during the subsequent intermittent exercise tests. The protocol replicated the seminal work by Vanhatalo et al. (2007), which involved 3-min unloaded pedalling into an abrupt start of the 3-min sprint phase, during which the ergometer instantly switched to the linear mode. A 10-s countdown was provided to prompt the participants to increase cadence over 120 rpm and to enable the attainment of peak power at the start of the sprint phase. Visual feedback of cadence was available during the test, with participants instructed to maintain the highest possible cadence throughout the test; strong verbal encouragement was provided for the whole test. The linear factor resistance (linear factor = power/cadence^2^) was established using the power output corresponding to 50% of the difference between VT1 and PPO during the RAMP test, along with the individual preferred cadences used during the ramp test. Critical power was calculated on the average power output during the last 30 s of the test and Wʹ as the volume of work (kJ) performed above CP. The test was deemed valid when there was no evidence of pacing; in that, the end 30-s power output was the lowest recorded throughout the test. In addition, pulmonary $\dot {V}$O_2_ had to reach 95% of maximum within 60 s of the sprint phase and be maintained without any downward drift for the remainder of the test (Jones et al. 2010). If these criteria were not attained, the test was repeated.

### Intermittent tests

All intermittent tests commenced with 1-min pedalling at 20 W followed by an abrupt start in to repeated intervals of 60-s work and 30-s recovery until exhaustion. The intensity of the work interval was determined by the intensity predicted to attain task failure in 4-min (P_4_), in accordance with the two-parameter CP model (Eq. 1), whilst the recovery was set at 20 *W*:

1 $${P_4}={\text{ }}\left( {W^{\prime}/240} \right){\text{ }}+{\text{ CP}}.$$

The use of threshold models to set the work profiles of intermittent exercise is suggested to increase the accuracy of standardising cardiopulmonary and metabolic response to exercise within and between individuals, relative to traditional percentiles of max heart rate, power output or $\dot {V}$O_2peak_ (Tschakert and Hofmann 2013). The work completed in the severe-intensity domain could also be calculated by multiplying the time spent above CP by the difference between the work intensity and CP.

A pre-established preferred cadence was held throughout the intermittent test, with task failure defined as the inability to maintain cadence within 60% of the preferred cadence (Amman et al. 2008), despite strong verbal encouragement. Exercise tolerance was determined as the time, in seconds, participants could maintain a cadence < 60% of their preferred cadence at the required power output; whilst the cumulative work performed above CP was defined as the work performed in the severe-intensity domain during the intermittent test. Heart rate (Forerunner 15, Garmin, US) and oxygen saturation (SpO_2_) using a fingertip pulse oximeter (Autocorr^®^ Digital Pulse Oximeter, BCI, US) were recorded at the end of every work stage and reported as a test average. Fingertip blood samples were collected prior to and within 1 min of exercise termination during intermittent experimental trials, using the methods described earlier. Samples were analysed for blood pH and [HCO_3_^−^] (Radiometer ABL800, Denmark), with blood lactate concentrations ([lactate]) assessed using a portable measuring device (Lactate Pro 2, Arkray, Japan).

### Statistical analysis

The Shapiro–Wilk test provided no evidence to reject the hypothesis that all data were normally distributed. A paired *t* test was used to compare the exercise durations, work completed in severe-intensity domain, mean heart rate and SpO_2_. Blood variables, including [HCO_3_^−^], [H^+^] and [lactate], were analysed through a two-way (treatment [placebo vs. NaHCO_3_] × time [pre-and post-exercise]) ANOVA. Where a significant main effect was found, Bonferroni post hoc paired comparisons were determined. Effect sizes and their 95% confidence intervals (CI) were calculated using Hedge’s *g* for paired comparisons, with the effects interpreted and discussed against effects of the relevant prior literature (Thompson 2007). These effect sizes can also be interpreted as trivial (< 0.20), small (0.20–0.49), moderate (0.50–0.79) or large (≥ 0.80) (Cohen 1988). The Hedge’s *g* correction was used to mitigate positive bias of the Cohen’s *d* effect size when using sample sizes less than 20 (Lakens 2013). Frequentist inferences were assessed against mean difference ± 95% CI between experimental conditions, with variances that do not cross the zero-boundary interpreted as significant. All descriptive data are presented as mean ± standard deviation, unless otherwise stated. Statistical analysis was performed using open source statistical software, R (R Foundation for Statistical Computing, Vienna, Austria).

## Results

**Fig. 1:**
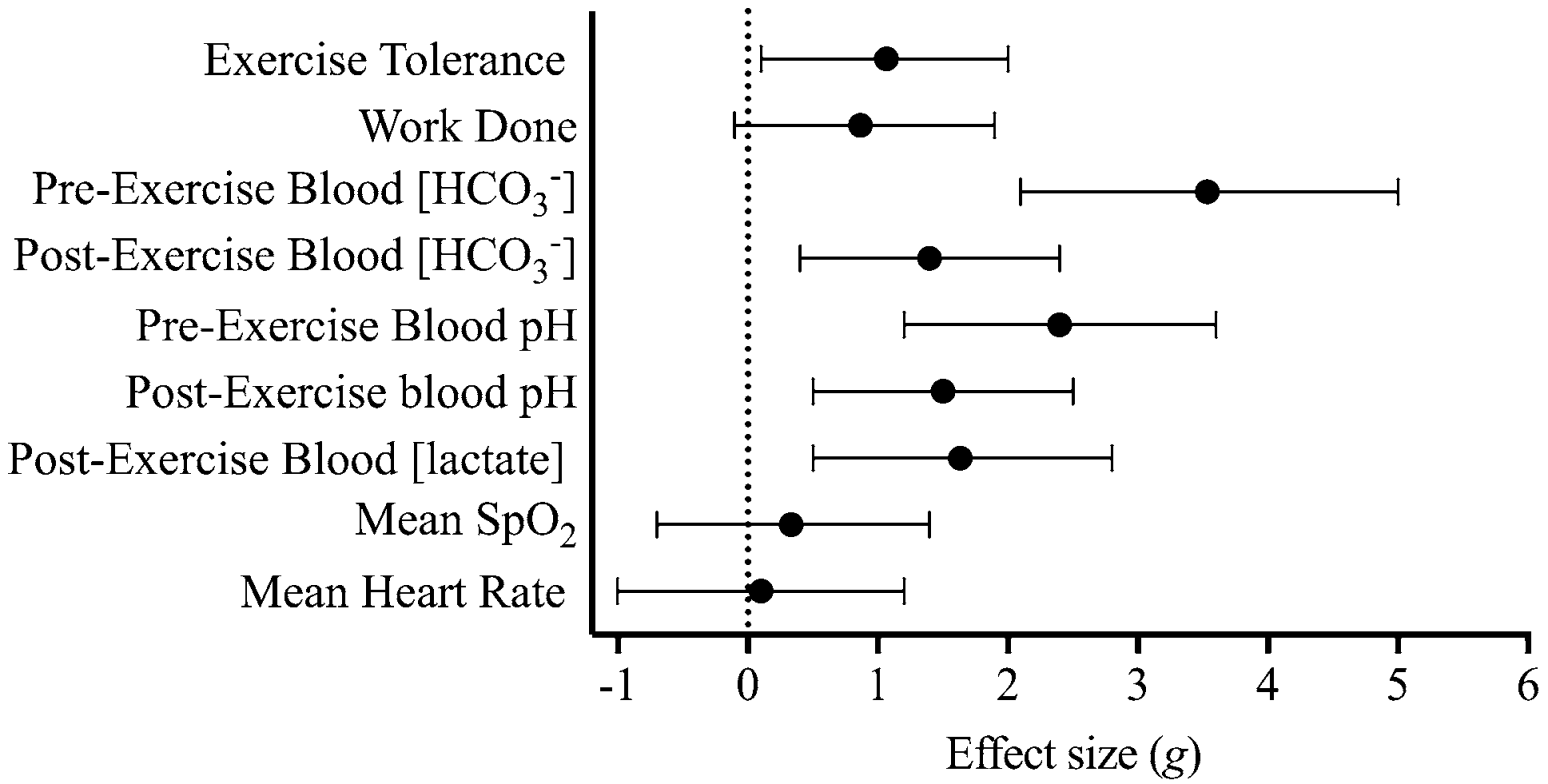
Hedge’s *g* effect size and 95% CI of the effect of NaHCO_3_ treatment against placebo treatment for all outcome variables

**Fig. 2:**
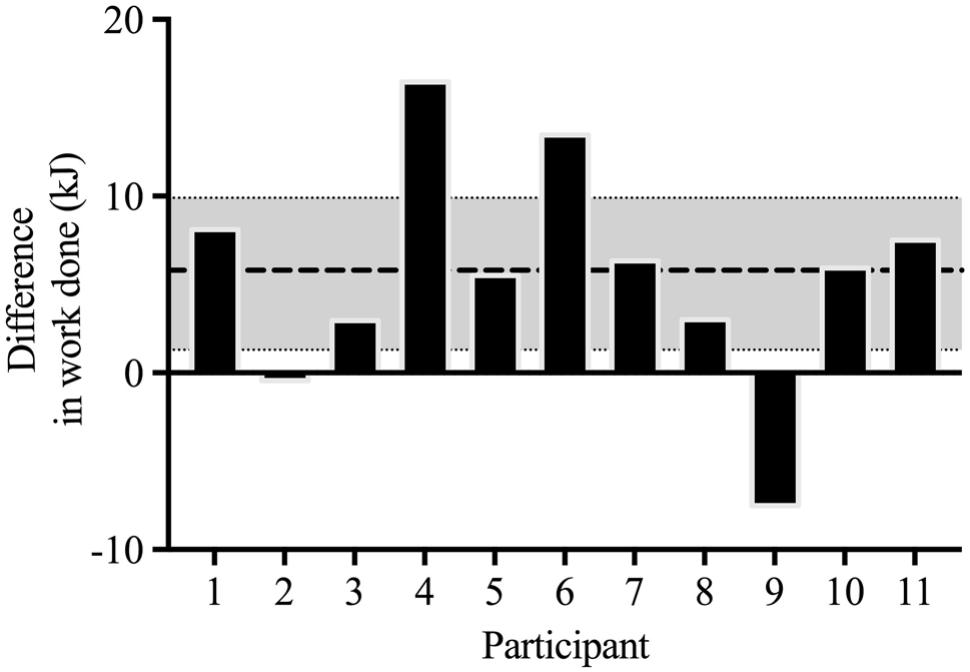
Difference in work complete between NaHCO_3_ treatment against placebo treatment for all participants. Values greater than zero indicate that a greater volume of work was performed with NaHCO_3_ and values lower than zero indicate that less work was performed with NaHCO_3_ compared to placebo. Dashed line represents mean difference in work complete and the shaded band shows the ± 95% CI of effect between treatments

The time taken to reach individual peak blood [HCO_3_^−^] ranged from 40 to 90 min with a median of value of 70 min. Mean CP and *W*ʹ were 226 ± 31 W and 20.3 ± 7.0 kJ, respectively. Exercise tolerance during the intermittent test (Fig. 1) was significantly greater during the NaHCO_3_ treatment condition by 14.6 ± 12.5% from 734.3 ± 175.7 s during the placebo condition to 845.3 ± 242.4 s under NaHCO_3_ experimental conditions (mean difference = 110.9 ± 100.6 s, 95% CI 43.3–178.5 s). Similarly, the work done in the severe-intensity domain was significantly increased by 5.8 ± 6.4 kJ (95% CI 1.3–9.9 kJ), from 41.6 ± 14.7 to 47.2 ± 17.6 kJ in the placebo and NaHCO_3_ conditions, respectively. Despite the positive outcome, an increase in work completed in the severe-intensity domain was not consistent across all participants, with participant 9 experiencing an ergolytic effect and participant 2 showing no difference between experimental conditions (Fig. 2).

**Fig. 3:**
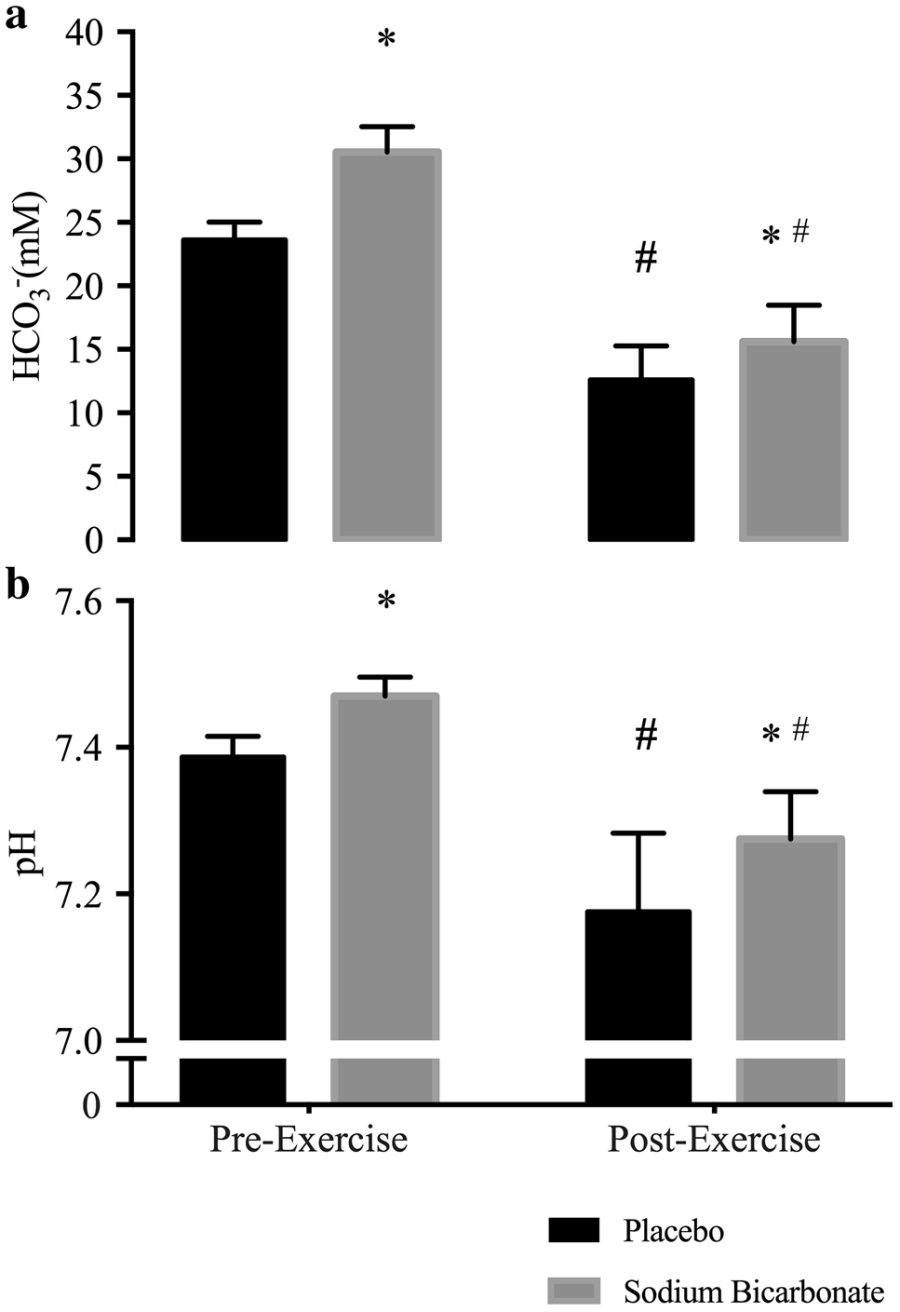
Pre- and post-exercise blood [HCO_3_^−^] (**a**) and blood pH (**b**) during NaHCO_3_ and placebo experimental trials. *Significant (95% CI) difference to corresponding placebo time point. ^#^Significant (95% CI) difference to corresponding pre-exercise time point within the same experiment condition

An overall main effect for blood [HCO_3_^−^] was apparent (Fig. 3a), with a significantly greater concentration observed with NaHCO_3_ compared to placebo prior to exercise (6.9 ± 1.9 mmol l^−1^; 95% CI 5.6–8.1 mmol l^−1^). Blood [HCO_3_^−^] remained elevated in the NaHCO_3_ treatment condition post-exercise compared to placebo (3.0 ± 2.0 mmol l^−1^; 95% CI 1.6–4.3 mmol l^−1^), despite the larger reduction in [HCO_3_^−^] during exercise in the NaHCO_3_ condition (− 14.9 ± 2.9 mmol l^−1^; 95% CI − 16.9 to − 13.0 mmol l^−1^) compared to placebo (− 11.0 ± 2.6 mmol l^−1^; 95% CI − 12.8 to − 9.3 mmol l^−1^). An equivalent result was found for pH (Fig. 3b), with a greater pre-exercise values observed in the NaHCO_3_ treatment condition compared to placebo (0.08 ± 0.03; 90% CI 0.06–0.11); whilst pH also remained elevated following exercise (0.09 ± 0.06 95% CI 0.05–0.15). This difference in pre- and post-exercise pH was apparent given the similar reduction during exercise in the placebo (− 0.21 ± 0.08; 95% CI − 0.28 to − 0.14) and NaHCO_3_ conditions (− 0.20 ± 0.08; 95% CI − 0.24 to − 0.15). Post-exercise blood [lactate] increased by 4.0 ± 2.4 mmol l^−1^ (95% CI 2.2–5.9) from 13.9 ± 4.3 mmol l^−1^ during the placebo condition compared to 17.9 ± 5.9 mmol l^−1^ with NaHCO_3_ treatment. There were no significant differences in mean heart rate (0.4 ± 4.7 bpm; 95% CI − 3.5 to 4.3 bpm) and SpO_2_ (0.8 ± 2.3%; 95% CI − 1.1 to 2.7%) between conditions.

## Discussion

The ergogenicity of NaHCO_3_ during intermittent exercise has only been demonstrated with work intervals involving maximal or supra-maximal intensity (Carr et al. 2011). Consequently, this study is the first to report that NaHCO_3_ improves exercise tolerance and work performed during intermittent exercise in the severe-intensity domain, whilst exposed to acute hypoxic conditions. Furthermore, this study demonstrated NaHCO_3_ improves severe-intensity intermittent exercise performance under acute hypoxic conditions. The blood acid–base and [lactate] perturbations during exercise observed in the current study are similar to previous investigations (Carr et al. 2011), therefore suggesting the ergogenic effects of NaHCO_3_ is likely to be mediated through the manipulation of acid–base balance. The current study also adds to the growing body of literature that has utilised a methodology to elicit peak blood [HCO_3_^−^] at the onset of exercise by accounting individual variance in blood acid–base kinetics following NaHCO_3_ ingestion (Miller et al. 2015; Deb et al. 2017). Together, this study demonstrates NaHCO_3_ supplementation prescribed to account for the individual variance in time to peak blood [HCO_3_^−^], enhances severe-intensity intermittent exercise under acute moderate hypoxic conditions. This therefore may offer an ergogenic strategy to improve high-intensity intermittent exercise tolerance under acute moderate hypoxic conditions.

This study adds to the paucity of research evaluating the effect of NaHCO_3_ supplementation on exercise performed in the severe-intensity domain, which have demonstrated equivocal outcomes (George and MacLaren 1988; Egger et al. 2014). Egger et al. (2014) reported a significant improvement equivalent to 0.41 Hedge’s *g* units in 21 well-trained cyclists; whereas George and MacLaren (1988) found no significant effect in seven healthy participants. This disparity between studies and the positive outcome in the current study, could be explained by a dose-dependent effect of NaHCO_3_. Given that the current study demonstrated positive outcomes with a relative 0.3 kg^−1^ body mass dose compared to the 0.2 g kg^−1^ body mass administered by George and MacLaren (1988). Interestingly, the observed effect on exercise tolerance and work done in the severe-intensity domain in current study was 2–2.5-fold greater than that previously observed during severe-intensity exercise. It is important to highlight the use of intermittent exercise in the current study and continuous exercise in previous research may also account for the larger ergogenic effect observed in exercise performance. A proposition that is supported with meta-analytic evidence, with Carr et al. (2011) reporting greater performance improvements with NaHCO_3_ during repeated compared to continuous exercise. Nevertheless, a hypoxic mediated effect cannot be dismissed, as the efficacy of NaHCO_3_ may be greater under acute moderate hypoxic conditions (Deb et al. 2017), due to the exacerbated acid–base perturbations (Hogan et al. 1999). This hypothesis should, however, be viewed with caution until further research has evaluated the efficacy of NaHCO_3_ with normoxic and hypoxic comparators.

Despite this assertion and the ergogenic outcomes found in the current study, a number of previous investigations have not reported similar performance-enhancing properties of NaHCO_3_ under acute moderate hypoxic conditions (Saunders et al. 2014a; Flinn et al. 2014). Flinn et al. (2014) performed a similar exhaustive intermittent exercise protocol to the present study, but utilised a supra-maximal exercise intensity under acute hypoxic conditions; while Saunders et al. (2014a) used a repeated sprint protocol prolonged across 90 min to simulate soccer performance, and found no positive performance effect. This discrepancy between the current study and prior research could be attributed to the timing of administering NaHCO_3_, since both Saunders et al. (2014a) and Flinn et al. (2014) used a gelatine capsule delivery method over a 4-h and 90-min period prior to exercise, respectively. In contrast, the current study accounted for the individual temporal characteristics of acid–base kinetics following NaHCO_3_ supplementation, by ensuring participants commenced exercise tests at the peak blood [HCO_3_^−^], to maximise buffering potential (Jones et al. 2016; Gough et al. 2017). Indeed, the time course to peak blood [HCO_3_^−^] using a gelatine capsule has previously been shown to range from 75 to 180 min and a median of 120 min in a similar participant cohort; while the range from liquid supplementation was between 40 and 90 min in the present study. However, when comparing pre-exercise blood [HCO_3_^−^] between current and previous studies it is not clear that utilising an individualised strategy is superior at maximising the ergogenic effect of NaHCO_3_. The current study reported a mean 6.9 mmol l^−1^(*g* = 3.5, 95% CI 2.1–5.0) increase in blood [HCO_3_^−^] compared to placebo, which is greater than the 5.7 mmol l^−1^ (*g* = 3.4, 95% CI 2.9–3.8) increase reported by Saunders et al. (2014a) but Flinn et al. (2014) reported a greater 7.6 mmol l^−1^ (*g* = 4.4, 95% CI 3.7–5.0) increase; despite showing no positive performance effect. Interestingly, the standardised effect sizes and corresponding 95% confidence intervals suggest the effect on pre-exercise [HCO_3_^−^] was similar between investigations as the confidence intervals overlap. Therefore, while an individualised supplementation strategy may be appropriate to maximised blood [HCO_3_^−^] prior to exercise within individuals (Jones et al. 2016; Gough et al. 2017); timing may not be the only residing factor that determines the ergogenicity of NaHCO_3_ supplementation. Further research is therefore required to determine the efficacy of individualised timing against a standardised timing strategy.

Despite the contemporary development of personalised NaHCO_3_ supplementation ingestion time, the adverse gastrointestinal (GI) side effects remain apparent. As evident in participant 9 (Fig. 2), who experienced substantial GI complaints, the side effects of supplementation may produce an ergolytic performance effect. Previous research suggests that the variability in NaHCO_3_ ergogenic properties may be dependent on presence and severity of GI symptoms (Saunders et al. 2014b). However, GI complaints and a negative or no performance effect are not always apparent, as the ergogenic and adverse effects of NaHCO_3_ have been shown to coexist (McNaughton et al. 2016), with Cameron et al. (2010) reporting a weak association between the two parameters (*r* = 0.35; *p* = 0.09). Given this lack of clarity in this relationship and the failure of the current study to empirically quantify GI complaints, the negative influence of NaHCO_3_ can only be inferred in this study as it provides little direct evidence to support the notion that GI complaints from NaHCO_3_ supplementation can impair exercise performance or lessen the ergogenic effects.

Independent of individual exercise performance effects, the temporal blood acid–base behaviour following NaHCO_3_ supplementation and exhaustive exercise, were comparable to the wider literature base (Carr et al. <link rid="bib9">2011</link; Flinn et al. 2014; Saunders et al. 2014a; Deb et al. 2017). Oral NaHCO_3_ supplementation induced peak blood [HCO_3_^−^] concentrations that are greater than 6 mmol l^−1^ compared to placebo, which is above the suggested level required for NaHCO_3_ to exhibit an ergogenic effect (Carr et al. 2011). The change in blood pH and [HCO_3_^−^] was greater during exercise with NaHCO_3_ compared to placebo, with an equivalent larger rise in blood [lactate] during the NaHCO_3_ experimental trial. Indeed, there is evidence to suggest NaHCO_3_ promotes non-oxidative energy metabolism, as observed through greater muscle lactate production, and muscle glycogen utilisation during intermittent exercise performed in the severe-intensity domain (Percival et al. 2015). Despite not measuring muscle glycogen utilisation or muscle lactate production in the current study, based on previous research it is appropriate to speculate that the observed ergogenic effects of NaHCO_3_ may be mediated through augmenting glycolytic bioenergetic contribution, and an enhanced muscle glycogen utilisation. While this is a plausible theory, it is important to highlight that the rise in blood [lactate] during the treatment condition may be explained by a reduction lactate uptake into inactive muscle tissue (Granier et al. 1996) and/or an increase in lactate efflux from intramuscular to extracellular regions (Bishop et al. 2004) following NaHCO_3_ supplementation. As such, an increase in glycolytic activity can only be speculated in the current investigation. In addition, it is also prudent to highlight that pH at the end of exercise did not reach comparable values in the experimental and placebo trials, which conforms with previous research (Carr et al. 2011). This suggests that pH may not be the solitary reason for exercise termination in the experimental condition. Given the multi-faceted nature of fatigue alternative explanations may exist; such as the strong ion difference, which refers to the intra- and extracellular ions (e.g. potassium, sodium, chloride) that are involved in skeletal muscle contraction. Evidence suggests NaHCO_3_ can alter the ionic charge of these compartments by attenuating the efflux of potassium ions from the musculature and therefore, maintaining muscle contractile properties during exercise (Siegler et al. 2016). Muscle potassium concentrations were however, not measured in the current study and consequently its role in this context can only be speculated. The SID also has an independent effect on pH (Stewart 1978), and thus it may exert skeletal muscle performance impairing effects through altering intra- or extra-cellular pH. Further research is required to understand the alternative reasons for exercise termination in the experimental condition despite pH remaining elevated; this may include assessing the function of the strong ion difference.

It is prudent to highlight the diversity in training status of the participant cohort as both trained and recreationally active individuals volunteered for this study. This does not however, limit the applicability of our findings given the wide-ranging applications of acute hypoxic training methods, from trained athletes (Faiss et al. 2013), healthy individuals (Shatilo et al. 2008) to patient cohorts (Millet et al. 2016). Consequently, further research investigating the effects of NaHCO_3_ as a training aid during acute hypoxic training programs in a range of population may be beneficial. In addition, a limitation to the application of this study may be in the environment at which NaHCO_3_ was ingested; in that, ingesting NaHCO_3_ whilst remaining under acute hypoxic conditions prior to exercise may have altered the outcomes of the study as opposed to the ingestion under normoxic conditions in this study. Indeed, exposure to hypoxic conditions is suggested to diminish blood [HCO_3_^−^] (Cerretelli and Samaja 2003) and therefore, prolonged prior hypoxic exposure may have perturbed the manipulation of the acid–base balance following NaHCO_3_ ingestion. This however, can only be hypothesised until further experimental work investigates the temporal acid–base response following NaHCO_3_ under acute hypoxic conditions and the subsequent impact on exercise performance. Nevertheless, this study is the first to demonstrate NaHCO_3_ supplementation in normoxic conditions can improve severe-intensity intermittent exercise performance under acute moderate hypoxic conditions. Therefore, providing a potential ergogenic strategy for individuals undertaking acute hypoxic exercise bouts as part of training programme. Furthermore, this study demonstrates that ingesting NaHCO_3_ at a pre-determined peak blood [HCO_3_^−^] prior to commencing exercise is an efficacious method to enhance blood HCO_3_^−^ buffering potential. However, caution should be taken as the adverse GI complaints associated with NaHCO_3_ may produce ergolytic effects. To build on the current investigation, further empirical research should consider the use of NaHCO_3_ as a training aid during hypoxic training strategies, to determine if repeated acute supplementation prior to exercise alters the molecular training adaptations associated with intermittent acute hypoxic training.

## Abbreviations

[HCO_3_^−^]: Bicarbonate anion concentrations
[lactate]: Blood lactate concentrations
CI: Confidence intervals
CP: Critical power
*g*: Hedge’s *g*
H^+^: Hydrogen cation
$\dot {V}$O_2peak_: Peak rate of oxygen consumption
O_2_: Oxygen
spo_2_: Oxygen saturation
NaHCO_3_: Sodium bicarbonate
NaCl: Sodium chloride
VT1: Ventilatory threshold 1
*W*ʹ: *W* prime
